# Conflict of Interest Policies for Organizations Producing a Large Number of Clinical Practice Guidelines

**DOI:** 10.1371/journal.pone.0037413

**Published:** 2012-05-22

**Authors:** Susan L. Norris, Haley K. Holmer, Brittany U. Burda, Lauren A. Ogden, Rongwei Fu

**Affiliations:** 1 Department of Medical Informatics and Clinical Epidemiology, Oregon Health and Science University, Portland, Oregon, United States of America; 2 Kaiser Permanente Center for Health Research, Portland, Oregon, United States of America; 3 Department of Public Health and Preventive Medicine, Oregon Health and Science University, Portland, Oregon, United States of America; The University of Adelaide, Australia

## Abstract

**Background:**

Conflict of interest (COI) of clinical practice guideline (CPG) sponsors and authors is an important potential source of bias in CPG development. The objectives of this study were to describe the COI policies for organizations currently producing a significant number of CPGs, and to determine if these policies meet 2011 Institute of Medicine (IOM) standards.

**Methodology/Principal Findings:**

We identified organizations with five or more guidelines listed in the National Guideline Clearinghouse between January 1, 2009 and November 5, 2010. We obtained the COI policy for each organization from publicly accessible sources, most often the organization's website, and compared those polices to IOM standards related to COI. 37 organizations fulfilled our inclusion criteria, of which 17 (46%) had a COI policy directly related to CPGs. These COI policies varied widely with respect to types of COI addressed, from whom disclosures were collected, monetary thresholds for disclosure, approaches to management, and updating requirements. Not one organization's policy adhered to all seven of the IOM standards that were examined, and nine organizations did not meet a single one of the standards.

**Conclusions/Significance:**

COI policies among organizations producing a large number of CPGs currently do not measure up to IOM standards related to COI disclosure and management. CPG developers need to make significant improvements in these policies and their implementation in order to optimize the quality and credibility of their guidelines.

## Introduction

Clinical practice guidelines (CPGs) are “statements that include recommendations intended to optimize patient care that are informed by a systematic review of evidence and an assessment of the benefits and harms of alternative care options,” according to the Institute of Medicine (IOM) [1]. CPGs can influence the care delivered by a large number of healthcare providers and thus the outcomes of patients [2], so their quality is critically important. High-quality guidelines have the potential to promote the use of effective clinical services, decrease undesirable practice variation, reduce the use of services that are of minimal or questionable value, increase the use of effective but underused services, and target services to populations most likely to benefit [3]. High-quality guidelines are valid (unbiased), reproducible, clinically applicable to the populations of interest, flexible, clearly presented, developed through a multidisciplinary process, reviewed on a regular schedule, and well-documented [3]. CPGs may improve processes of care [2], [4], [5], however data on the effectiveness of CPGs on health outcomes are sparse and conflicting [1], [2], [4]–[6].

Conflict of interest (COI) is an important potential source of bias in the development of CPGs. A COI is a set of conditions in which professional judgment concerning a primary interest (such as the health and well being of a patient or the validity of research), is unduly influenced by a secondary interest [7]. There are data that suggest an association between author or funder COI and study outcomes [8]–[11] and between COI and systematic review conclusions [12]. Data on the prevalence of industry relationships of guideline sponsors and authors are limited and dated, however [13]–[16], and evidence of the effect of these relationships on guideline recommendations is confined to case studies [17]. Nonfinancial interests may also be powerful motivators for guideline authors [18], including the advancement of medical science, career advancement, fulfillment of a desire to do good, opportunity to publish, notoriety, future success in obtaining funding for research, and increased sense of self worth [19].

Recent IOM reports highlight the importance of COI in healthcare [20], systematic reviews [21], and CPGs [1], and provide standards for disclosure and management of COI. The extent to which current CPG organizational policies align with IOM standards has not yet been reported. The objective of this study was to describe the COI policies of organizations currently producing a large number of CPGs, to compare these policies to the recent IOM standards related to COI in CPG development [1], and to identify characteristics of organizations that correlate with policies that meet these standards.

## Methods

We identified organizations that had five or more guidelines listed in the National Guideline Clearinghouse (NGC) (http://www.guideline.gov) that were published between January 1, 2009 and the date of our search (November 5, 2010). We selected this cohort in order to examine the policies of organizations that have the potential for a major effect on physician behavior and patient outcomes. The NGC, funded by the Agency for Healthcare Research and Quality (AHRQ), was established in January of 1999, “to provide physicians and other health professionals, health care providers, health plans, integrated delivery systems, purchasers, and others an accessible mechanism for obtaining objective, detailed information on clinical practice guidelines and to further their dissemination, implementation, and use” [22]. The inclusion criteria for guidelines within the NGC are:

1. The CPG contains systematically developed statements that include recommendations, strategies, or information that assists physicians and/or other health care practitioners and patients to make decisions about appropriate health care.
2. The CPG was produced under the auspices of medical specialty associations; relevant professional societies, public or private organizations, government agencies at the Federal, State, or local level; or health care organizations or plans.
3. Corroborating documentation can be produced and verified that a systematic search and review of existing scientific evidence was performed during the guideline development.
4. The full text guideline is available upon request in the English language.
5. The guideline is current, in that it was developed, reviewed, or revised within the last 5 years.

Two authors (HKH, BUB) independently searched for each organization's COI policy on the organization's website and through a general internet search. Only policies that were specific to CPG development were examined further: the policy was required to either include a statement specifically regarding CPGs or be included in a CPG development handbook, and at a minimum address COI for CPG panel members. Policies that covered a variety of organizational activities were not examined further because such general policies did not address adequately address the issues of COI relevant to CPG development. If the guideline developer was US-based, the authors also determined if the CPG organization was signed onto the Council of Medical Specialty Societies' (CMSS) Code for Interactions with Companies, by examining the CMSS website [23]. CMSS, a collaboration of almost 40 US-based medical specialty organizations, issued a revised code in March, 2011 to provide guidance for members on how to minimize and manage conflicts of industry that arise from their relationships with companies that produce drugs, devices, and other healthcare interventions. Underpinned by the principles of independence and transparency, the CMSS code provides specific guidance for the conduct of educational programs, business interactions, receipt of funding support, sponsorship, and advocacy, among others.

We did not include information contained within the CPG or in the summary provided by the NGC as the former was invariably insufficient to assess the organization's policy and the latter was information provided by the organization to the NGC after the guideline was completed. We did not contact organizations for additional information, nor did we try to obtain information from organizations with protected websites for members only, because we wanted to include only information that was publicly available to users of CPGs, and our past experiences trying to obtain unpublished COI policies suggested that such an approach was not likely to be productive.

One author abstracted information on each organization's COI policy into a pre-specified template in Excel®, and those data were checked by a second author. The data abstracted from each policy included when disclosures were obtained, from whom, over what time period, the types of COI (financial or intellectual), and how disclosures were managed. Data were synthesized across guidelines using descriptive statistics and a qualitative synthesis. We used a logistic regression model to explore the association between whether an organization had a CPG-specific COI policy and pre-specified predictors included type of organization, the number of CPGs produced between 2009 and our search date, and whether or not the organization was based in the US.

We compared the COI policy of each guideline organization to the COI-related standards in the 2011 IOM report (Table 1) [1] for the purpose of describing the degree to which organizations are already meeting these standards and the gaps that need to be addressed. We selected the IOM standards as a comparator for our cohort of guidelines because this report is the most recent, large-scale compendium of evidence and recommendations for guideline development in healthcare that we are aware of. We are aware of extensive efforts across North American guideline groups to implement these standards. We assessed the seven IOM standards that were deemed evaluable at the level of the organizational policy (standards 2.1, 2.2a, 2.2b, 2.3, 2.4a, 2.4c, 2.4d). We did not assess standards that were best assessed at the level of the CPG (standard 1.1) or standards that were unlikely to be included in an organization's COI policy (standard 2.4b, 2.4e).

**Table 1:** Institute of Medicine standards for developing trustworthy clinical practice guidelines (2011)[1].

IOM Standard
1.1: * The process by which a CPG is developed and funded should be detailed explicitly and be publicly accessible.
2.1:† Prior to selection of the CPG group, individuals being considered for membership should declare all interests and activities potentially resulting in COI with development group activity, by written disclosure to those convening the guideline development group. Disclosure should reflect all current and planned commercial, noncommercial, intellectual, institutional, and patient/public activities pertinent to the potential scope of the CPG.
2.2a: All COI of each GDG member should be reported and discussed by the prospective development group prior to the onset of his or her work.
2.2b: Each panel member should explain how his or her COI could influence the CPG development process or specific recommendations.
2.3: ¥ Members of the GDG should divest themselves of financial investments they or their family members have in, and not participate in marketing activities or advisory boards of, entities whose interests could be affected by CPG recommendations.
2.4a: Whenever possible, GDG members should not have COI.
2.4b:* In some circumstance, a GDG may not be able to perform its work without members who have COI, such as relevant clinical specialists who receive a substantial portion of their income from services pertinent to the CPG.
2.4c: Members with COI should represent not more than a minority of the guideline development group.
2.4d: The chair or co-chairs should not be a person(s) with COI.
2.4e:* Funders should have no role in CPG development.

Abbreviations: COI, conflict of interest; CPG: clinical practice guideline; GDG, guideline development group.

(*) We did not assess this standard as it would not be included in a COI policy.

† An organizations' COI policy must include all listed criteria in order to meet this standard in Table 5.

¥ Not applicable to COI policies that exclude any COI among guideline panel members.

Three authors (SLN, HKH, BUB) independently made a determination as to whether each organization's policy met each IOM standard and then consensus was achieved through discussion. Inter-rater reliability was assessed by using Kappa statistics for each standard and overall for the seven standards combined.

We then explored pre-specified predictors for organizations fulfilling these IOM standards, using the same variables that we used in our examination of whether each organization had a COI policy or not. The association between meeting each standard or at least one standard and the predictors was assessed using a logistic regression model. The association between meeting any IOM standard and the predictors was assessed using a logistic generalized estimating equation (GEE) model, accounting for correlation among multiple standards within each organization. The association between the proportion of standards met in each organization and the predictors was explored using a linear regression model.

## Results

### Characteristics of Organizations Producing CPGs

596 CPGs were published in NGC between 2009 and the date of our search in 2010, by 118 organizations listing at least one guideline. Of these organizations, 37 fulfilled our inclusion criteria, publishing 392 guidelines (66% of the total of 596 CPGs) (Table 2). The majority of the 37 organizations (86.5%) published fewer than 20 guidelines, while one organization published more than 40 [24]. Professional (56.7%) and government (29.7%) organizations made up the majority of the 37 organizations.

**Table 2:** Characteristics of included organizations (n = 37).

Organization	Number of CPGs*	Type of organization	Country	COI policy for CPGs	Effective date of COI policy	Signed on to CMSS†
Academy of Breastfeeding Medicine (ABM)	5	Professional	US	Yes	2010	Yes
American Academy of Neurology (AAN)	7	Professional	US	Yes	2009	Yes
American Academy of Pediatric Dentistry (AAPD)	5	Professional	US	No	NA	No
American Academy of Sleep Medicine (AASM)	5	Professional	US	No	NA	No
American Association for the Study of Liver Diseases (AASLD)	9	Professional	US	Yes	2010	No
American College of Foot and Ankle Surgeons (ACFAS)	6	Professional	US	No	NA	No
American College of Obstetricians and Gynecologists (ACOG)	19	Professional	US	Yes	2011	Yes
American College of Radiology (ACR)	21	Professional	US	No	NA	No
American Diabetes Association (ADA)	11	Professional	US	Yes	INF	No
American Optometric Association (AOA)	8	Professional	US	No	NA	No
American Society for Parenteral and Enteral Nutrition (ASPEN)	7	Professional	US	No	NA	No
American Society of Anesthesiologists (ASA)	8	Professional	US	No	NA	No
American Society of Plastic Surgeons (ASPS)	6	Professional	US	No	NA	No
American Urological Association (AUA)	7	Professional	US	Yes	2011	Yes
Cancer Care Ontario (CCO)	17	Government	Canada	No	NA	NA
Centers for Disease Control and Prevention (CDC)	13	Government	US	No	NA	No
Cincinnati Children's Hospital Medical Center (CCHMC)	32	Healthcare Provider	US	No	NA	No
European Association of Urology (EAU)	52	Professional	Netherlands	Yes	INF	NA
European Society for Paediatric Urology (ESPU)	17	Professional	UK	No	NA	NA
Infectious Diseases Society of America (IDSA)	8	Professional	US	No	NA	No
Institute for Clinical Systems Improvement (ICSI)	25	Consortium‡	US	Yes	2007	No
Michigan Quality Improvement Consortium (MQIC)	13	Consortium§	US	Yes	2007	No
National Collaborating Centre for Mental Health (NCC-MH)	5	Government	England	Yes	2009	NA
National Collaborating Centre for Women and Children's Health (NCC-WCH)	6	Government	England	Yes	2009	NA
National Institute for Clinical Excellence (NICE)	34	Government	England	Yes	2009	NA
New York State Department of Health (NYDOH)	13	Government	US	No	NA	No
New Zealand Ministry of Education (NZMOE)	7	Government	New Zealand	No	NA	NA
New Zealand Ministry of Health (NZMOH)	7	Government	New Zealand	No	NA	NA
Ontario Ministry of Health and Long-Term Care (OMHLTC)	17	Government	Canada	No	NA	NA
Program in Evidence-based Care (PEC)	17	Professional	Canada	Yes	INF	NA
Registered Nurses' Association of Ontario (RNAO)	5	Professional	Canada	Yes	INF	NA
Scottish Intercollegiate Guidelines Network (SIGN)	5	Government	Scotland	Yes	2008	NA
Society of Obstetricians and Gynecologists of Canada (SOGC)	16	Professional	Canada	Yes	2003	NA
U.S. Preventive Services Task Force (USPSTF)	13	Government	US	Yes	2008	No
University of Michigan Health System (UMHS)	5	Academic	US	No	NA	No
Washington State Department of Labor and Industries (WSDLI)	5	Government	US	No	NA	No
Work Loss Data Institute (WLDA)	5	Industry	US	No	NA	No
**Summary (N = 37)**	461	Professional: 21 (56.7%)	US: 24 (64.9%)	Yes: 17 (45.9%)	Range, 2003–2011	Yes: 4 (16.7% of US-based organizations)
	5–9: (N = 21) 57%	Government: 11 (29.7%)			Median 2009	
	10–14: (N = 5) 14%	Academic: 1 (2.7%)			INF: 4 (23.5%)	
	15–19: (N = 6) 16%	Industry: 1 (2.7%)				
	>20: (N = 5) 14%	Other: 3 (8.1%)				

* Number of CPGs published between January 1, 2009 and November 5, 2010 (search date).

† As identified on the CMSS website (http://www.cmss.org/OriginalSigners.aspx).

‡ Consortium sponsored by five state health plans (payers) and comprised of 62 medical groups (providers).

§ Consortium of healthcare providers, payers and specialty societies.

Abbreviations: CMSS: Council of Medical Specialty Societies; COI: conflict of interest; CPG: clinical practice guideline; INF: information not found; NA: not applicable.

### Conflict of Interest Policies

Seventeen of the 37 organizations (45.9%) had a COI policy that was publicly accessible and applicable to the development of CPGs (Table 3). The remaining organizations either did not have a policy or it was not publicly available (43.2%), or the only identified policy was not explicitly related to CPG development (10.8%). Organizational characteristics were not significantly associated with the presence of a COI policy, including the production of fewer CPGs (p = 0.24), governmental compared to a professional organization (p = 0.65), and based in the US compared to other countries (p = 0.17).

**Table 3:** Organizational policies on financial and non-financial conflict of interest (n = 17).

Organization	Policy definition of COI	Types of financial COI	Financial threshold	Non-financial COI addressed	Types of non-financial COI	Precludes industry funding	Relevance mentioned
ABM [25]	All financial and uncompensated relationships with companies	S	NR	Yes	I	Yes	Yes
AAN [29]	A financial stake in the success or failure of the products appraised in the CPG	C, S, P, E, O	NR*	Yes	I	Yes	Yes
AASLD [26]	If a person or their immediate family might gain financially or non-financially from the actions of the person while acting on behalf of AASLD. If a person who serves AASLD also serves another organization, whose mission overlaps that of AASLD, in a capacity that can potentially put that organization in direct competition with AASLD thereby impeding the ability of AASLD to fulfill its mission. Conflicts of interest are further defined as any circumstances that create a risk that professional judgment or actions regarding a primary interest will be unduly influenced by a secondary interest. Primary interests are those associated with the stated mission of the AASLD. Secondary interests may be financial or non-financial in nature	C, S, P, E, G, O	$5,000	Yes	V	Yes	No
ACOG [27]	Not defined	C, S, P, E, G, O	$25–$5,000†	No	NA	No	No
ADA [38]	Duality of interest: individuals have material interests outside the ADA that could influence them or could be perceived as influencing them to act contrary to the interests of the ADA and for their own personal benefit or that of a family member or a business associate	C, S, E	NR	Yes	I	No	No
AUA [28]	Relationships or associations with organizations, persons, corporations or enterprises that may affect or be perceived to affect one's judgment or decision-making	C, S, P, E, O	NR	Yes	V	Yes	No
EAU [24]	Situations involving a duality of interest which might be interpreted as COI	NA (financial COI not addressed specifically)	NA	No	NA	No	No
ICSI [30]	Any potential conflict and competing interests with any organization with commercial, proprietary, or political interests relevant to the topics covered by ICSI	O	NR	No	NA	No	No
MQIC [31]	Relationships with pharmaceutical companies, biomedical device manufacturers, or other corporations whose products or services are related to the guideline subject matter	C, S, E, G, O	NR	No	NA	No	No
NCC-MH [32]NCC-WCH [33]NICE [34]	A personal pecuniary interest involves a current personal payment, which may either relate to the manufacturer or owner of a product or service being evaluated, in which case it is regarded as ‘specific’ or to the industry or sector from which the product or service comes, in which case it is regarded as ‘non-specific’	C, S, P, E	NR	Yes	V, I	No	Yes
PEC [43]	Not defined	O	NR	No	NA	No	No
RNAO [44]	Not defined§	NA	NA	No	NA	No	No
SIGN [35]	Specific interests are those which relate to a topic or remit of the particular guideline. Non specific interests are those which are otherwise relevant to the work of SIGN. For their partners or close relatives, interests are restricted to employment in, or share holdings in, healthcare organizations	C, S, E, G, O	NR	Yes	NR	No	No
SOCG [37]	Breach of an obligation that has the effect or intention of advancing one's own interest or the interests of others in a way detrimental to the interests, or potentially harmful to the public or the integrity and fundamental mission of the SOGC	C, G	NR	Yes	V	No	No
USPSTF [36]	Each corporation, company, firm, research organization, educational institution, or other organization or institution (proprietary and not-for-profit, domestic and foreign) in which you, your spouse, and dependent children have significant financial interests that are related to the subject matter	C, S, P, E	$10,000 or >5% ownership‡	Yes	I	No	Yes

* Financial COI declared in ranges (less than $10,000, $10,000 to $25,000, and greater than $25,000).

† Having a financial interest of greater than $5,000 in an individual pharmaceutical company or an individual manufacturer of medical instruments, devices or equipment; acceptance of anything with a value greater than $25 for their personal use from an individual pharmaceutical company or an individual manufacturer of medical instruments, devices or equipment.

‡ Required to disclosure amounts greater than $10,000 per year or as determined through reference to public prices or other reasonable measure of fair market value, or represents more than 5% ownership interest in any single entity.

§ RNAO requires that all members declare COI in writing during recruitment or selection of guideline panel members, but no other information is provided in their policy.

Key to types of conflict of interest: C: paid consultancy or speaking engagement. S: research or salary support. P: patent or royalties. E: equity or stock. G: gifts. O: other (e.g., travel grants). I: non-financial or intellectual (not otherwise specified). V: opinion or viewpoint.

Abbreviations: COI, conflict of interest; NA, not applicable; NR, not reported; see Table 1 for abbreviations for the guideline organizations.

Twelve [25]–[36] of the 17 organizations (71%) with a COI policy implemented their policy within the last 5 years; one organization [37] had a policy dating back to 2003. Four organizations [25], [27]–[29] signed onto the CMSS Code of Interactions with Companies [23], however, only one organization [25] acknowledged their CMSS affiliation website and within their COI policy.

COI was variably defined across the 17 policies (Table 3), of which 15 included the terms “financial” or “commercial” [24]–[38]. Disclosure of specific financial relationships varied among organizations: the most common types were paid consultancies, research or salary support, patents or royalties, equity or stock, and gifts. Only three organizations [26], [27], [36] specified a dollar-value threshold for required reporting of a financial interest.

The types of non-financial COI described in the policies also varied (Table 3). Eleven organizations (65% of 17) specifically required that intellectual or other non-financial COI be disclosed [25], [26], [28], [29], [32]–[38]. Four of these organizations did not provide a definition for non-financial COI, but rather used terms such as “intellectual” or “other related” conflicts [25], [29], [35], [38]. The remaining seven organizations provided more detailed, and very heterogeneous, descriptions [26], [28], [32]–[34], [36], [37]. One organization required the disclosure of competing beliefs, academic institutions, societies and publications [28], while another organization required the reporting of substantial career efforts (effort devoted to procedures currently performed in clinical practice) [29]. One organization [26] used the definition of intellectual COI as proposed by Guyatt and colleagues [18] where an attachment to a specific viewpoint as developed through academic and other activities could unduly affect an individual's judgment about a specific guideline recommendation.

### Disclosures

All organizations required disclosure from all guideline development group members (Table 4). The editorial board (5.8%), systematic review authors (5.8%), expert reviewers (17.6%), and other guideline contributors (5.8%) such as expert advisors and staff were also specifically required to disclose in some policies. Seven organizations (23.5%) required that the disclosure policy extend to relatives or dependents of guideline development group members [26], [28]–[30], [35], [36], [38].

**Table 4:** Disclosure requirements and conflict of interest management strategies for organizations (n = 17).

Organization	About whom is COI collected	When is initially COI collected	Time period for disclosure	Policy on updating COI	Management strategy goes beyond disclosure*	Management strategy	Who assesses COI disclosures	Who determined plan of action based on disclosures	Penalties for non-disclosure
ABM	CPG panel members (including chair), expert reviewers	Prior to deliberations	Past 12 m	Updated throughout guideline development process	Yes	Will not appoint individual with COI as CPG panel chair; majority of CPG panel will not have COI	NR	NR	NR
AAN	CPG panel members (including chair)	NR	Past 60 m	Updated annually and at time of CPG publication	Yes	May not appoint individual with COI as chair or lead author of CPG if COI deemed significant (i.e., above thresholds); no current industry employees allowed on panel	Advisory body	Advisory body	May be subject to discipline
AASLD	CPG panel members (including chair), expert reviewers	Change in role	Past 12 m	NR	Yes	After review of COI, individual with COI may be asked to divest/resign, abstain from discussion or voting, excuse self from participant, or resign from CPG panel	CPG committee	Ethics committee	NR
ACOG	College committee members	NR	NR	NR	No	NR	NR	NR	Reported to president and vice president
ADA	Contributors of ADA publications (including authors, editors and editorial board members)	At least on appointment	NR	NR	Yes	Individual with COI must refrain from any discussion or action related to subject including abstention from voting	Other committee	Other committee; board of directors	NR
AUA	CPG panel members (including chair), expert reviewers, expert consultants	Prior to participation	Past 12 m	NR	Yes	May lead to recusal or exclusion from participation	Committee chairs	Judicial and ethics committees	Termi-nation/expulsion, removal from panel and censorship
EAU	CPG committee members	NR	NR	NR	No	Not applicable (individuals with COI are not involved in CPG development)	NR	NR	NR
ICSI	CPG developers	NR	NR	NR	No	NR	NR	NR	NR
MQIC	CPG developers	NR	NR	NR	Yes	Disclosure and judged by committee if bias present	Medical director's committee	Medical director's committee	NR
NCC-MH; NCC-WCH; NICE	CPG panel members (including chair)	At least on appointment	Past 12 m	NR	Yes	Individuals with COI excluded from CPG panel, participation on advisory board may be allowed	Committee chair	Advisory body	NR
PEC	CPG workgroup members (including chair)	NR	NR	NR	No	NR	NR	NR	NR
RNAO	CPG panel members (including chair)	Recruitment or selection of panel	NR	NR	No	NR	NR	NR	NR
SIGN	Committee members	NR	Past 12 m	NR	No	NR	NR	NR	NR
SOCG	Committee members	NR	NR	NR	Yes	COI does not preclude participation however position may be vacated	NR	NR	NR
USPSTF	CPG members (including chair), systematic review staff	Prior to deliberations (at least triennially), at least on appointment	Past 12 m	NR	Yes	Individual with COI may have no action (e.g., recusal), may fully participate as lead, recalls from participant from lead but allowed to vote, or complete recusal	Funder	CPG chair and/or vice-chair(s); funder	NR

* Disclosure management strategy goes beyond simply requiring clinical practice guideline members to disclose conflicts of interest (COI). Management strategies include, for example, descriptions of who assesses and/or manages the disclosures, penalties for non-disclosure, detailed decision-making processes, limitations placed on individuals with COI, or prohibition from participation.

Abbreviations: COI, conflict of interest; NR, not reported; see Table 2 for abbreviations of the guideline organizations.

Disclosures are collected at various time points across the organizations, (Table 4) most commonly at the time of appointment to a guideline panel or prior to beginning deliberations. Two organizations addressed updating of COI, either throughout the CPG process [25] or just prior to CPG publication [29]. The reporting period for COI ranged between the prior 12 to 60 months (median 12 months).

Seven organizations (41.2%) limited the disclosure to only those that were relevant to the content of the CPG (Table 2) [25], [28], [29], [32]–[34], [36]. In most organizations relevance was not defined, however, although three organizations that shared a common COI policy require disclosure for personal payments, “which may either relate to the manufacturer or owner of a product or service being evaluated, in which case it is regarded as ‘specific’ or to the industry or sector from which the product or service comes, in which case it is regarded as ‘non-specific’ ” [32]–[34].

### Management of Disclosures

Eleven organizations (64.7% of 17) delineated strategies to manage COI after disclosure, with considerable variation in these approaches (Table 4). Advisory panels (5.8%), the CPG committee (23.5%), committee chairs (5.8%), or another party (e.g., funder of the CPG) (17.6%) assessed the disclosures. In some cases the same body of individuals assessed and managed the disclosures (17.6%). More commonly, however, a party separate from those involved in the assessment of disclosures devised a management plan: advisory panel (17.6%), the CPG chair (5.8%), or an ethics committee (11.8%). For 47% of organizations, a significant conflict resulted in recusal from the CPG development group or abstention from the final recommendation process. One policy detailed penalties for non-compliance with the organization's policy [28], while two other organizations stated that non-compliance would lead to disciplinary action [27], [29].

### IOM Standards for Reporting Conflicts of Interest in Clinical Practice Guidelines

None of the 17 organizations with a CPG-specific COI policy met all seven IOM standards specific to COI (Table 5). The policies of eight organizations [25], [26], [28], [29], [32]–[34], [36], [38], however, met at least one of the seven standards, with five organizations meeting at least four [25], [29], [32]–[34]. The standard most frequently met was 2.1 (n = 7) and 2.2a (n = 8). None of the organization characteristics was statistically significantly associated with meeting any standard, including number of CPGs listed in NGC by that organization in our 2009 to 2010 cohort (p = 0.21); professional organization compared to government sponsor (p = 0.81); and US-based organization versus other countries (p = 0.85). Regression models examining these same predictors for meeting each individual standard, at least one standard, as well as the proportion of standards met for each organization were also not significant (p<0.05).

**Table 5:** Organizational policies and the Institute of Medicine standards [1].

IOM Standard	SOCG	MQ I C	S I GN	ABM	AAN	ACOG	I CS I	US P S TF	NCC-MH	NCC-WCH	N I CE	AAS LD	AUA	ADA	EAU	PEC	RNAO	No. (%) meeting standard
Effective date of policy	1999	2003	2003	2006	2006	2007	2007	2008	2009	2009	2009	2010	2011	INF	INF	INF	INF	
2.1: Prior to selection of panel members, individuals provide COI disclosures	N	N	N	N	Y	N	N	N	Y	Y	Y	Y	Y	Y	N	N	N	7 (41%)
2.2a: COI should be discussed before CPG work starts	N	N	N	Y	Y	N	N	N	Y	Y	Y	Y	Y	Y	N	N	N	8 (47%)
2.2b: Each member should explain how their COI could influence the CPG	N	N	N	N	N	N	N	N	N	N	N	N	N	N	N	N	N	0 (0%)
2.3: Divestment by members	N	N	N	N	N	N	N	N	N	N	N	Y	N	N	N	N	N	1 (6%)
2.4a: When possible, members should not have COI	N	N	N	Y	Y	N	N	N	Y	Y	Y	N	N	N	N	N	N	5 (29%)
2.4c: Members with COI should make up a minority of panel members	N	N	N	Y	Y	N	N	N	N	N	N	N	N	N	N	N	N	2 (12%)
2.4d: Chair(s) and co-chair(s) should be free of COI	N	N	N	Y	Y	N	N	N	Y	Y	Y	N	N	N	N	N	N	5 (29%)
Number (%) of standards met	0 (0%)	0 (0%)	0 (0%)	4 (57%)	5 (71%)	0 (0%)	0 (0%)	0 (0%)	4 (57%)	4 (57%)	4 (57%)	3 (43%)	2 (29%)	2 (29%)	0 (0%)	0 (0%)	0 (0%)	28 (23%)

Each cell depicts whether or not the organization's policy met all of the criteria for each standard.

Abbreviations: COI, conflict of interest; INF, information not found; NR, not reported; see Table 2 for abbreviations for the guideline organizations.

The agreement among the three assessors for all seven IOM standards across all 17 policies was 94.1% (95% CI, 88.3% to 97.6%). Agreement for specific standards ranged from 76.5% (standard 4a) to 100.0% (standards 2b, 3, 4d). The overall Kappa statistic for inter-rater reliability among the three reviewers was 0.88.

## Discussion

Fewer than half of organizations that have recently listed a large number of guidelines in the NGC have a COI policy directly related to CPGs. Of organizations with such policies, these vary widely with respect to types of COI addressed, from whom disclosures are collected, monetary thresholds for disclosure, approaches to management, and updating requirements. Not one organization's policy adhered to all seven of the IOM 2011 standards applicable to COI policies for CPGs, and approximately half of these organizations do not adhere to a single one of these standards.

There is a need for extensive improvements in COI policies for organizations producing CPGs. Every developer must have a COI policy specific to CPGs: a general COI policy for the organization or a policy that relates to research subjects, for example, is not adequate to address the issues of secondary interests relevant to guideline development. Guidelines involve multiple groups of individuals and processes, and the COI of all of these must be disclosed: authors of the systematic review, the guideline panel members, and other individuals involved in the formulation and approval processes. COI policies for CPGs must also address management of the disclosures, starting with the requirement that the guideline panel chair and co-chair have no relevant COI.

There are important implications of our findings for users of CPGs. Given the evidence that financial relationships of researchers are associated with study findings [8]–[11], [39] and that guideline panel composition correlates with panel recommendations [40], the reader needs to have ready access to accurate and up-to-date disclosures, and be assured that secondary interests have been appropriately managed.

Nonfinancial COI may be an important source of bias, and two-thirds of organizations make some mention of this type of COI in their policies. Definitions are variable, however, and often vague and difficult to interpret, potentially leading to inconsistent and incomplete reporting. The current knowledge base on the impact of nonfinancial COI on decision making is sparse, so that the development of evidence-based guidance on the disclosure and management of nonfinancial COI is difficult at the present time.

The IOM standards related to COI do not address all of the important issues for CPG developers and users. In particular, these standards do not address accessibility of COI policies, the relevance of secondary interests to the primary interest, and the optimal presentation of disclosures. We often encountered a great deal of difficulty locating COI policies: these policies should be readily accessible from the guideline organization's website. Policies on disclosures should address both relevance and an appropriate level of detail. Inadequate guidance within COI policies on these issues may reflect lack of evidence on both the relationship between specific types of conflicts and risk of bias, and what constitutes optimal disclosures for the reader. IOM standards also do not address accountability for accurate disclosures and adherence to the policy within the organization. In other words, adherence to these seven IOM standards does not ensure an optimal COI policy.

There are limitations to our approach. Most importantly, we did not assess if and how these policies were applied during CPG development and publication. Nor did we examine the impact of each organization's policy on their processes, disclosures, recommendations, and the use and interpretation of the guidelines by healthcare providers. Our descriptive work provides the basis for these important next steps in evaluating the nature and effect of COI policies in CPG development.

It is possible that our summary of these organizations' policies is not up to date. Given the current attention to COI policies by biomedical journals [41], [42] and organizations producing CPGs [1], [23], the COI policies for the organizations that we examined may have changed between development of the CPGs listed on the NGC website, our examination of the policy, and the present. Our information on specific policies may be incomplete in spite of having two independent persons identifying COI policies due to difficulty locating policies in various locations in linked websites.

We did not contact organizations for additional, unpublished information, nor did we try to obtain information from organizations with protected websites. We felt that if the guideline was in the public domain the COI policy should be there also, given the presumed importance of transparency in COI processes and disclosures. We also encountered challenges in determining if an organization's COI policy was specific to CPGs. Two independent reviewers made this assessment and then came to consensus, but it is possible that some of the organizations that we assessed as not having a CPG-specific policy would consider themselves to have such a policy. We would counter that these policies need to be more transparent and to address the processes and individuals involved in CPGs specifically.

The applicability of our findings to other organizations producing CPGs may be limited. The COI policies of organizations producing a small number of CPGs (less than 5) may differ from the policies that we examined. In addition, we focused only on CPGs published in English and those listed in NGC: the policies of other organizations may differ from our findings. The IOM standards were developed by a US-based group of experts, and thus encompass the American perspective on guideline topics, methodology, available resources, and the standards target U.S. patient populations and health care providers. The evidence base examined in the IOM report encompasses studies from a variety of international settings, and thus the IOM standards are likely applicable to guideline development in the Western world.

The IOM standards [1] were released after the publication of the CPGs examined herein. Thus our goal in comparing the IOM standards to existing organizational polices was to describe the current status of these policies and to identify areas where improvements are needed and not to criticize organizations for not having met these standards. Our reporting of the gaps in current approaches may aid organizations in planning updates of their COI policies and in comparing policies across organizations.

The IOM standards were not intended to be a quality assessment tool, thus implementing them as such was problematic. Standard 2.1 addresses both financial and nonfinancial COI, thus the two types of COI are not distinguished. Many of the standards are too vague to implement as quality assessment criteria: for example, standard 2.4a (Table 1).

COI policies among organizations producing a large number of CPGs currently do not measure up to IOM standards. Policy-makers, guideline funders, sponsors, and developers, as well as users need to address and demand improvements. Patients and populations need trustworthy CPGs, and the accurate disclosure and subsequent management of COI is essential to achieve that goal.
